# Synergistic effects of Mediterranean diet combined with phytosterol-based supplements and omega-3 fatty acids on lipid profiles: a pilot study in menopausal women

**DOI:** 10.3389/fnut.2025.1645102

**Published:** 2025-09-11

**Authors:** Fabiana Cannella, Elisa Assunta Algaria, Kashi Brunetti, Simona Del Quondam, Diego Bottan, Davide Cervia, Elisabetta Catalani

**Affiliations:** ^1^Department for Innovation in Biological, Agro-Food and Forest Systems (DIBAF), Università degli Studi della Tuscia, Viterbo, Italy; ^2^ASST Fatebenefratelli Sacco Hospital, Milan, Italy

**Keywords:** menopause, dyslipidemia, cholesterol, Mediterranean diet, supplement

## Abstract

**Objectives:**

Menopause marks the cessation of ovarian function, preceded by perimenopause, a transitional phase characterized by hormonal fluctuations and metabolic changes, including dyslipidemia. Therefore, a targeted nutritional approach is essential. In this retrospective, observational, pilot study, we evaluated the impact of a Mediterranean-based dietary regimen supplemented with specific natural compounds on lipid profiles and body composition in perimenopausal and menopausal women with hypercholesterolemia.

**Methods:**

An individual dietary plan based on the Mediterranean diet, supplemented with a phytosterol-based formula containing bergamot, prickly pear extract, and vitamin B1, was recommended for each study participant. Additionally, omega-3 fatty acid supplementation was recommended due to its well-documented benefits in reducing cardiovascular disease risk factors, including elevated lipid levels. Lipid profile, body composition, and anthropometric values were recorded and carefully analyzed.

**Results:**

Our findings indicated that this combined dietary approach significantly improved lipid profiles, as evidenced by reductions in total cholesterol, low-density lipoprotein cholesterol, and triglycerides and by an increment in high-density lipoprotein cholesterol values. Furthermore, the dietary plan positively impacted overall body composition and morphometric parameters.

**Conclusion:**

These preliminary findings suggest that a personalized, nutritionally targeted approach may be an effective non-pharmacological strategy for managing cardiovascular and metabolic risk factors during the menopausal transition and postmenopausal period. Further large-scale, controlled studies are warranted to confirm these results and explore long-term outcomes.

## 1 Introduction

Menopause is clinically defined as the absence of menstrual periods for 12 consecutive months, with the cessation of ovarian function (1, 2). Menopause typically begins between the ages of 45 and 55, and it is preceded by perimenopause, a transitional phase during which the ovaries gradually reduce the production of key reproductive hormones, triggering symptoms that may persist in the menopausal period. Starting from the early stages of the menopausal transition, women frequently experience a variety of disorders, including weight gain, increased visceral fat, insulin resistance, dyslipidemia, and hypertension, which enhance the risk of cardiovascular disease (CVD) (1). Hyperlipidemia is a key factor associated with the menopausal transition and menopause, involving changes in the levels of apolipoproteins, low-density lipoproteins (LDLs), high-density lipoproteins (HDLs), and triglycerides (TGs) in the bloodstream (3). In particular, high TGs, total cholesterol (TC), LDL, and a high TC-to-HDL ratio could be observed. In this scenario, hormone therapy offers some long-term health benefits; however, it is not indicated for preventing CVD (2, 4). Therefore, a proper lifestyle is crucial, including enhancing nutrition (1). Indeed, a healthy dietary pattern can support the maintenance of a healthy body condition, alleviating menopause symptoms and improving overall health throughout life (5). In this respect, a recent study showed that natural oral supplements containing glucosinolates, phytosterols, and citrus flavonoids were more efficacious in reducing global, physical, and psychosocial menopausal symptoms in the short term than estrogen plus progestogen therapy (6). Additionally, supplementation with vitamin D, omega-3 fatty acids, antioxidants, and phytochemicals supports the effects of a healthy nutritional regimen (7). Thus, recent evidence supports the use of personalized nutritional approaches for alleviating menopausal symptoms (1). The Mediterranean diet (MD) and lifestyle, characterized by anti-inflammatory and antioxidant-rich foods, offer significant health benefits (8–10), and alleviate menopausal symptoms (11, 12). To some extent, the MD significantly impacts health as a medical prescription in obese menopausal women (11).

Mediterranean regions are particularly rich in foods that can be utilized as functional foods. This is the case of bergamot (*Citrus bergamia Risso, family Rutaceae)*, an endemic plant native to the Calabrian region in southern Italy, that is rich in flavonoids and glycosides, which exhibit a wide range of pharmacological activities (13). Bergamot extract helps manage lipid levels (9, 11), and positively influences metabolic syndrome (14). Multiple studies indicate that the bergamot content of polyphenols, particularly flavonoids, is responsible for its beneficial effects on lipid profile (15–18). Noteworthy, a positive impact on lipid profile was reported in individuals after administering a nutraceutical compound based on a flavonoid complex from bergamot and opuntia (19). Opuntia ficus-indica (prickly pear, a member of the Cactaceae family), widely distributed in the Mediterranean region, is rich in pectins, mucilages, polyunsaturated fatty acids, and vitamins. Its supplementation has been shown to reduce CDV risk and positively affect blood lipids (20–22). Adherence to a dietary plan is a crucial issue in weight management and metabolic health (23). Albeit the adherence to the MD is decreasing due to the increased consumption of refined, processed food (24), we aimed to support the benefits of a specific dietary plan based on the Mediterranean pattern, in conjunction with specific supplements that potentially increase its efficacy, as a strategy to rebalance dyslipidemia during the menopausal transition and menopausal period. In particular, in this retrospective, observational, pilot study, we analyzed routinely collected data from a cohort of women in the perimenopausal and menopausal periods, who were affected by hypercholesterolemia, and followed a dietary regimen to improve their lipid profiles. Women were recommended to follow a personalized diet plan based on the MD concept, supplemented with a phytosterol-based formula containing bergamot, prickly pear extract, and vitamin B1. Since omega-3 fatty acids lessen CVD risk factors (25), diet supplementation with omega-3 fatty acids was also recommended.

During a few months of observation, individuals were monitored for lipidemic parameters, and in parallel, for anthropometric and body composition measurements. The data collection highlighted the positive effects of our dietary interventions in rebalancing the lipidemic profile and overall body size and composition, thus supporting the feasibility of larger-scale trials. Therefore, this pilot observational study was primarily intended to establish a basis for more comprehensive and rigorous future investigations.

## 2 Methods

### 2.1 Study design, participants, and ethics

This pilot study consisted of the observational retrospective analysis of data from a case series of 14 women, 9 menopausal and 5 perimenopausal, who attended the professional practice of a nutrition specialist and required dietary intervention due to dyslipidemia and weight management. The inclusion criteria were: TC > 200 mg/dL, LDL-C > 100 mg/dL, and a body mass index (BMI) between 18.5 and 39.9 kg/m^2^. The exclusion criteria included comorbidities, pharmacological treatment aimed at improving lipid profiles, and supplement intake (even if not explicitly targeting lipid levels). All participants provided written informed consent for the anonymous use of their data for research purposes at the end of the observational period. The study was conducted following the principles of the Declaration of Helsinki. In line with national regulations and due to the non-interventional, observational nature of the research involving fully anonymized data, ethical committee approval was not required. To note, the study did not collect or process data that could identify the participants or compromise their privacy.

All participants were of the same ethnicity and resided in the same Mediterranean area. A preliminary clinical interview was conducted to assess dietary habits and lifestyle, allowing the formulation of personalized nutritional plans tailored to individual needs. Nearly identical questions were administered to all participants by the nutritionist and systematically assessed at the initial evaluation and each subsequent follow-up.

This is an observational, open-label study, with no randomization of participants.

### 2.2 Data collection

Data collection took place between 2023 and 2024, beginning with the recruitment phase and followed by monthly check-ins. The duration of follow-up varied between individuals, ranging from a minimum of 2 months to a maximum of 7 months, depending on each participant’s clinical progress. This follow-up period variation reflects the frequency of parameters that require follow-up outside the nutritionist’s area of expertise (e.g., blood tests), as indicated by the attending physician, based on each participant’s progress. Furthermore, the notable improvements in lipid profiles, observed as early as two months after the initial assessment, support the rationale behind this specific aspect of the study.

Anthropometric measurements and body composition analyses and basal metabolic rate were performed using bioelectrical impedance analysis (Akern srl, Italy). The level of physical activity was estimated during the initial interview by determining the Metabolic Equivalent of Task (MET) value, a physiological measure that expresses the intensity of physical activities (26, 27). Menopausal women were found to be sedentary, while perimenopausal women were engaging in light physical activity. The metabolic rate and MET value were used to calculate the daily energy requirement for each participant. Biochemical parameters, including TC, HDL-C, LDL-C, and TG, were obtained from routine blood tests carried out independently by each participant at certified laboratories, based on recommendations provided by their general practitioners.

### 2.3 Dietary regimen

Dietary schemes were developed using commercial software (Progeo Medical^®^ Software, Italy), and based on the 2018 revision of the Guidelines for a Healthy Diet, which is known for its effectiveness in managing lipid profiles and weight (28, 29), developed by the Council for Agricultural Research and Economics Analysis (CREA) - an agency that operates under the supervision of the Italian Ministry of Agriculture, Food Sovereignty and Forests. It is recommended to consume five balanced meals daily: breakfast, lunch, dinner, and two snacks. Suggested foods should be indicated by their raw weights and net of waste, and possible substitutions should be specified. Extra-virgin olive oil should be considered as a condiment fat. Furthermore, fats should be 25%–35% of total calories, with saturated fats <7% of total calories, polyunsaturated fats up to 10% of total calories, monounsaturated fats up to 20% of total calories, and cholesterol <200 mg/day. Based on these general recommendations and women’s lifestyle, age, and the level of physical activity, the nutritionist recommends a personalized dietary plan with an average daily caloric intake of 1,378 kcal for menopausal women and 1,479 kcal for those in perimenopause. In particular, a 500-kilocalorie (kcal) deficit was applied from the estimated daily energy requirement, allowing for a well-tolerated and healthy body weight loss (30). Specifically, macronutrient intake is structured as follows: 1.5 grams of protein per kilogram of lean body mass, 0.6 grams of fat per kilogram of total body weight, and carbohydrates are calculated based on the remaining caloric needs, with a daily intake of 20–30 grams of fiber.

During the observational period participants intake two tablets of an omega-3 supplement containing EPA (300 mg), DHA (200 mg), and vitamin E (2.5 mg) after the three main meals (breakfast, lunch and dinner) and one tablet of a phytosterol-based supplement containing phytosterols (400 mg), bergamot (200 mg), prickly pear (150 mg), and vitamin B1 (12.5 mg) per tablet after dinner. Adherence to the diet and supplement intake was self-reported.

### 2.4 Statistics

The sample size of the experimental group was evaluated using G*Power 3 software (31). The power analysis indicated that a sample size of 13 items would provide 80% power to detect a substantial effect size (Cohen’s *d* = 0.85) at an alpha level of 0.05. In our study, a high effect size is expected based on the characteristics of typical blood tests and anthropometric measures, which are highly standardized, reliable, and subject to minor periodic variations. Therefore, the sample size of this study is adequately powered to detect the effect, although it is at the limit of the recommended size. Raw data were analyzed by comparing the final follow-up (t_*f*_) available and initial evaluation (t_0_) values of each participant, for each parameter under investigation, using the paired *t*-test (considering a *p*-value ≤ 0.05 as statistically significant). The 95% confidence intervals (95% CI) were set for all estimates. Data from different individuals were then normalized and averaged in the same graph using GraphPad Prism 6 software (GraphPad Software, San Diego, CA, USA). Results were expressed as the mean ± SEM of the indicated *n*-values.

## 3 Results

### 3.1 Characterization of participants and personalized dietary plan approach

In this study, we examined data collected from a group of women in the menopausal (mean age 60.1 ± 2.7 years; Supplementary Table 1) or perimenopausal (mean age 52.8 ± 1.6) phase with dyslipidemia who followed a dietary plan and supplementation as part of routine nutritional care. The key markers, including TC, LDL-C, HDL-C, and TG values, used to assess the lipid profile, were recorded during a follow-up period based on the results of serum analyses prescribed by the primary care physician. Therefore, the t_*f*_ for these values corresponds to a time window ranging from 2 to 7 months.

At t_0_, the average BMI of menopausal and perimenopausal participants was 26.8 ± 3.7 kg/m^2^ and 27.8 ± 3.7 kg/m^2^, respectively (Supplementary Table 1). Therefore, most participants started with a BMI in the overweight or obesity range (32).

The women’s lipid profile (Supplementary Table 1) revealed elevated TC and LDL-C levels and low HDL-C levels. In addition, 22% of menopausal women displayed elevated plasma TG levels.

**FIGURE 1:**
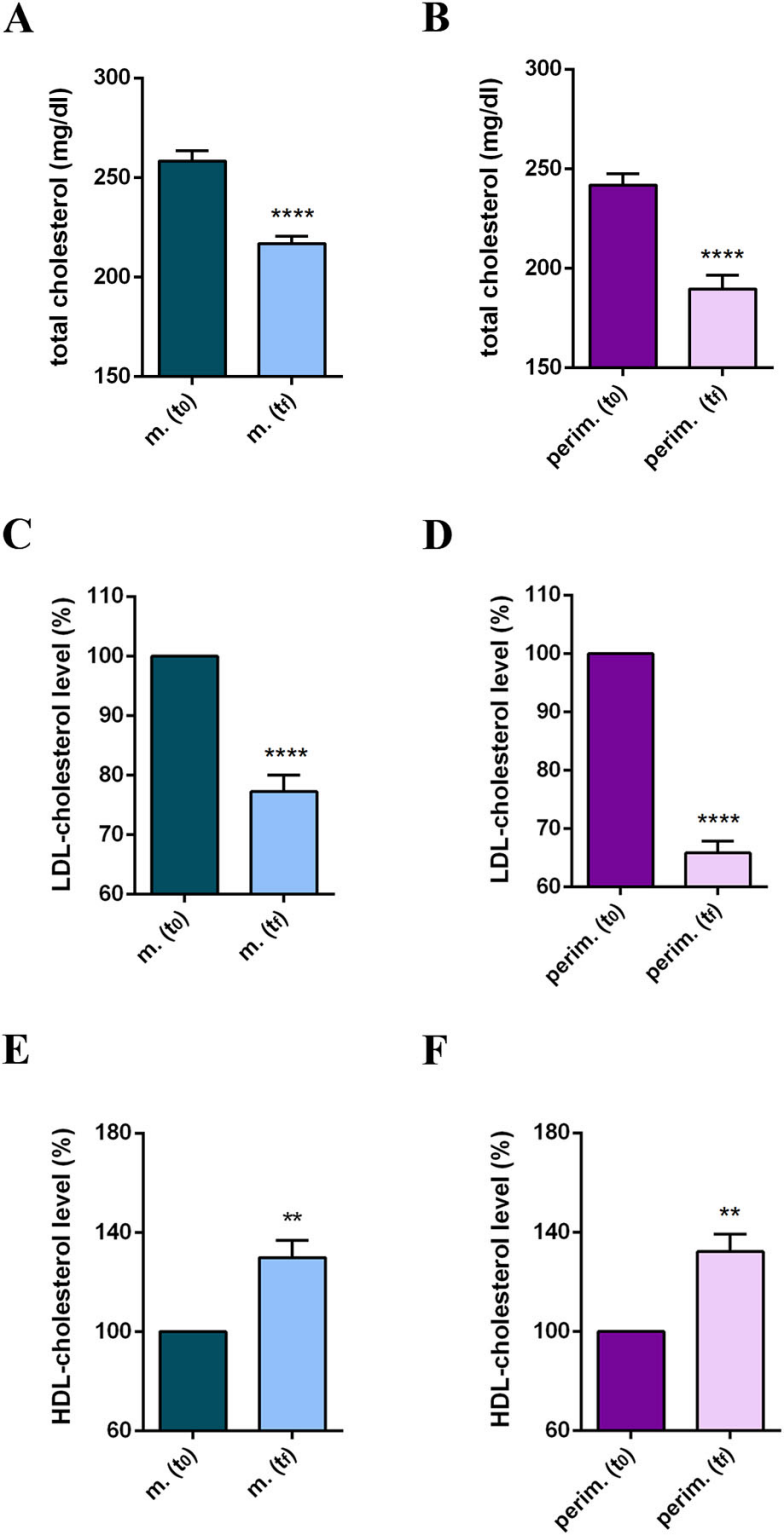
Cholesterol profile of m. (menopausal; *n* = 9) and perim. (perimenopausal; *n* = 5) groups at t_0_ (initial evaluation) and t_*f*_ (final follow-up). Total cholesterol (mg/dl) levels of m. **(A)** and perim. **(B)** groups. LDL (low-density lipoprotein)-cholesterol levels in m. **(C)** and perim. **(D)** groups. HDL (high-density lipoprotein)-cholesterol levels in m. **(E)** and perim. **(F)** groups expressed as the percentage of the t_0_ group. Raw data were analyzed using the paired *t*-test (considering a *p*-value ≤ 0.05 as statistically significant). **(A,B)** 95% CI = [–49,55 to –33,34] and [–60,07 to –44,33], respectively. **(C,D)** 95% CI = [–54,55 to –29,41] and [–72,26 to –52,06], respectively. **(E,F)** 95% CI = [7,257 to 21,41] and [7,622 to 21,18], respectively. **p < 0.01 and ****p < 0.0001 vs t_0_.

**FIGURE 2:**
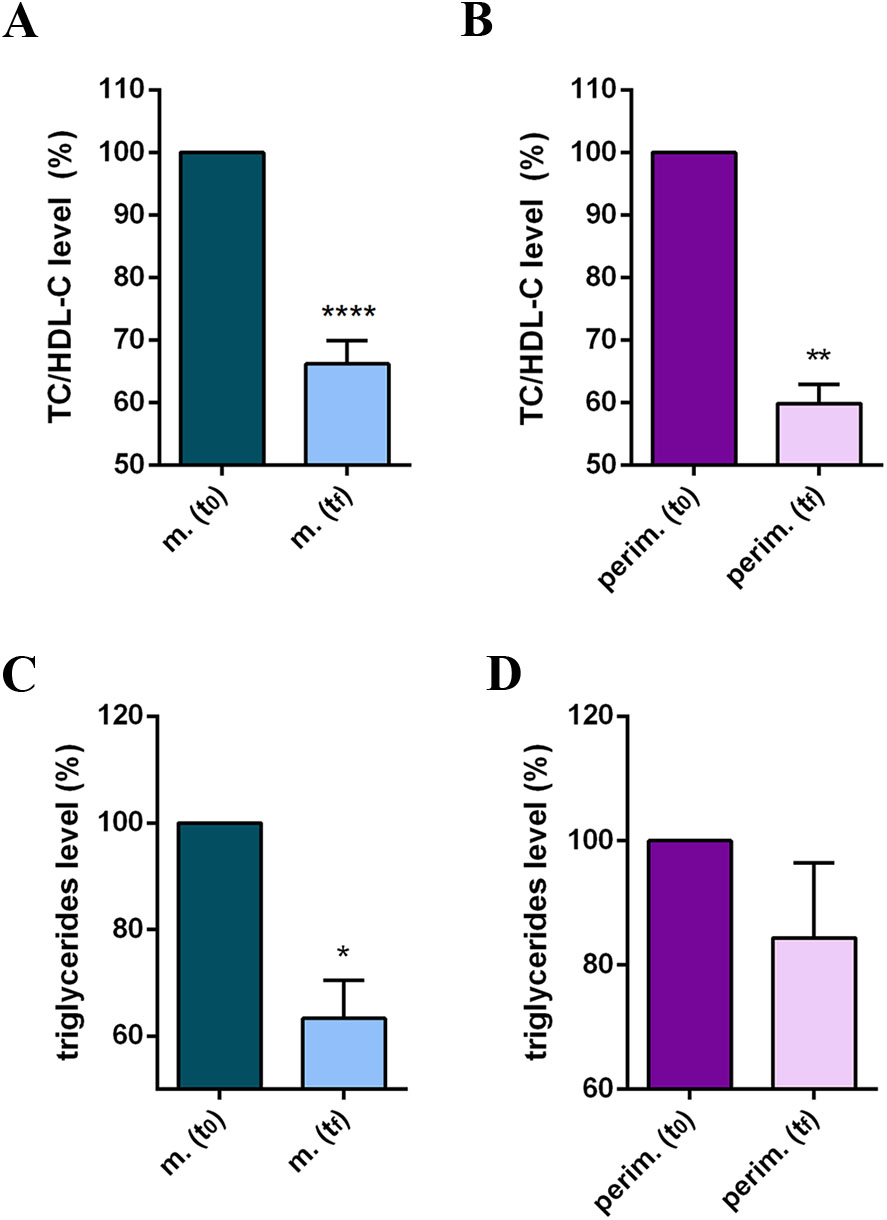
Lipid profile of m. (menopausal) and perim. (perimenopausal) groups at t_0_ (initial evaluation) and t_*f*_ (final follow-up). TC (total cholesterol)/HDL-C (high-density lipoprotein-cholesterol) ratio of m. **(A)** and perim. **(B)** groups. Triglyceride levels in m. **(C)** and perim. **(D)** groups. Raw data were analyzed using the paired *t*-test (considering a *p*-value ≤ 0.05 as statistically significant). **(A,B)** 95% CI = [–2,31 to –1,26] and [–2,94 to –1,40], respectively. **(C,D)** 95% CI = [–100,7 to –10,61] and [–56,09 to 22,89], respectively. *p < 0.05, **p < 0.01, ****p < 0.0001 vs t_0_.

Participants’ daily diet was modified according to the MD pattern, and the nutritionist incorporated omega-3 and phytosterol-based supplements into the routine plan. Results at t_*f*_ reflected a positive and promising response to dietary intervention and showed improved cholesterol profiles in all participants in the study (Supplementary Table 2). Statistical analysis of pooled data revealed a significant reduction in TC (Figures 1A, B) and in the LDL-C levels (Figures 1C, D), and a significant increase in HDL-C levels (Figures 1E, F) in both the two menopause phases. The HDL-C increment, up to 65% in menopausal women and up to 58% in perimenopausal women (Supplementary Table 2), appeared particularly relevant because the threshold of 50 mg/dl is considered a diagnostic marker for metabolic syndrome and a risk factor for CVD. Based on this evidence, the TC/HDL-C ratio and plasma TG levels were further analyzed (Supplementary Table 3). All participants at t_0_ had a TC/HDL-C ratio above or close to 4.5, possibly associated with an elevated CVD risk. By the t_*f*_, most participants had lowered their TC/HDL-C ratios to values below 4.0, achieving the target for primary prevention. Statistical analysis confirmed a significant and robust reduction in the TC/HDL-C ratio in menopausal and perimenopausal women (Figures 2A, B, respectively). Furthermore, a significant decrease in TG levels in the menopausal group (Figure 2C) was revealed. On the contrary, no difference was achieved in perimenopausal participants (Figure 2D), probably due to data variability within this group.

### 3.2 Anthropometric and body composition benefits following a personalized dietary plan and supplementation

The benefits of the dietary interventions were then assessed by anthropometric and body composition measurements and analysis (Supplementary Table 4).

**FIGURE 3:**
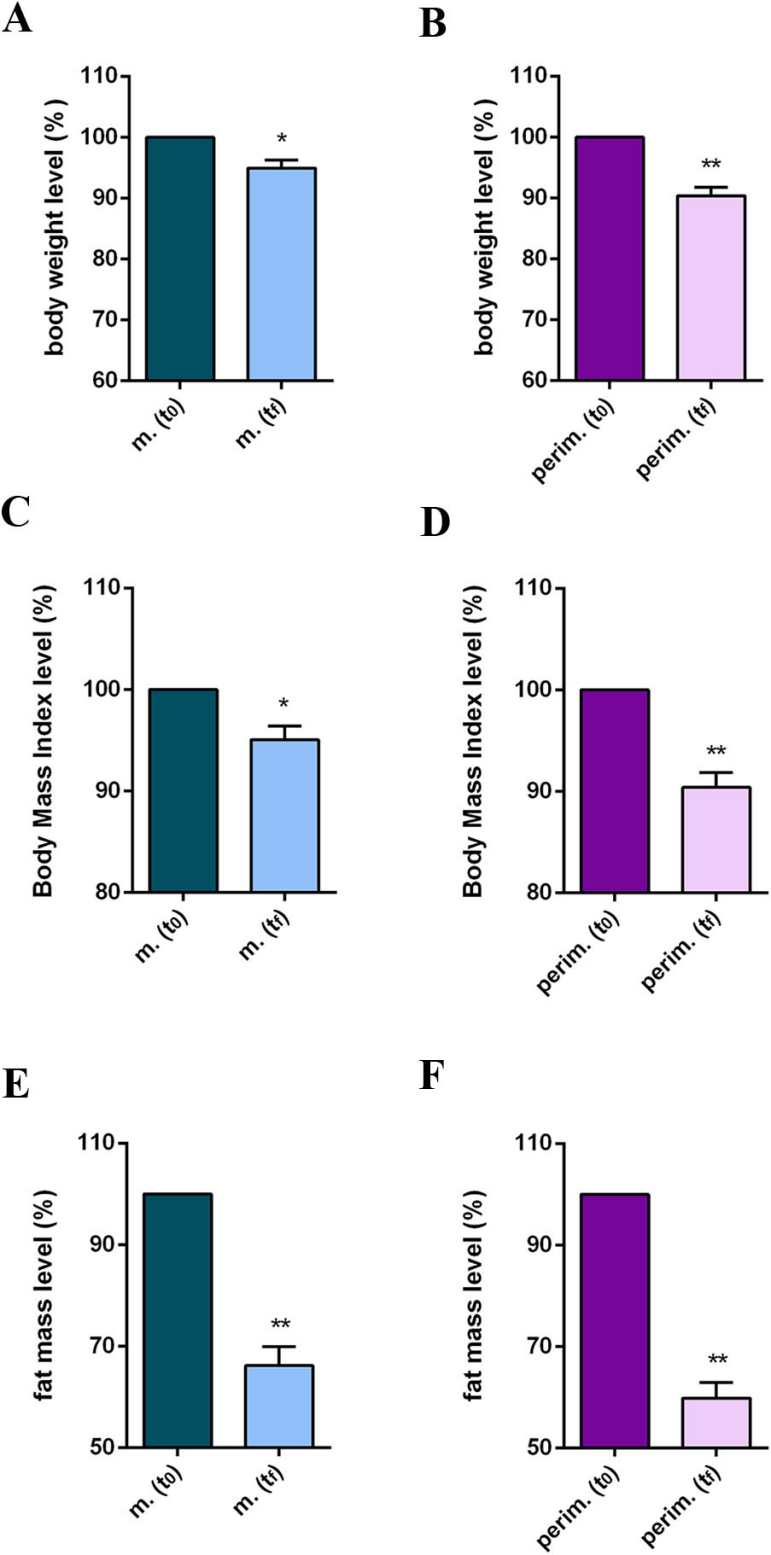
Anthropometric and body composition levels of m. (menopausal) and perim. (perimenopausal) groups at t_0_ (initial evaluation) and t_*f*_ (final follow-up). Body weight levels of m. **(A)** and perim. **(B)** groups. Body mass index levels in m. **(C)** and perim. **(D)** groups. Fat mass levels in m. **(E)** and perim. **(F)** groups. Raw data were analyzed using the paired *t*-test (considering a *p*-value ≤ 0.05 as statistically significant). **(A, B)** 95% CI = [–6,71 to –0,27] and [–11,19 to –3,49], respectively. **(C,D)** 95% CI = [–2,59 to –0,39] and [–4,23 to –1,29], respectively. **(E,F)** 95% CI = [–6,55 to –2,00] and [–10,39 to –3,89], respectively. *p < 0.05, **p < 0.01 vs t_0_.

The observed progress in BMI values is noteworthy, although not all women reached the healthy range of values. Additionally, a consistent fat mass (FM) reduction of up to 28% in menopausal and 32% in perimenopausal women was observed. Interestingly, FM reduction was also observed in a woman who failed to lose weight and had an overweight BMI. Statistical analysis confirmed a significant body weight (BW; Figures 3A, B), BMI (Figures 3C, D), and FM decrement (Figures 3E, F) in the two menopausal phases.

**FIGURE 4:**
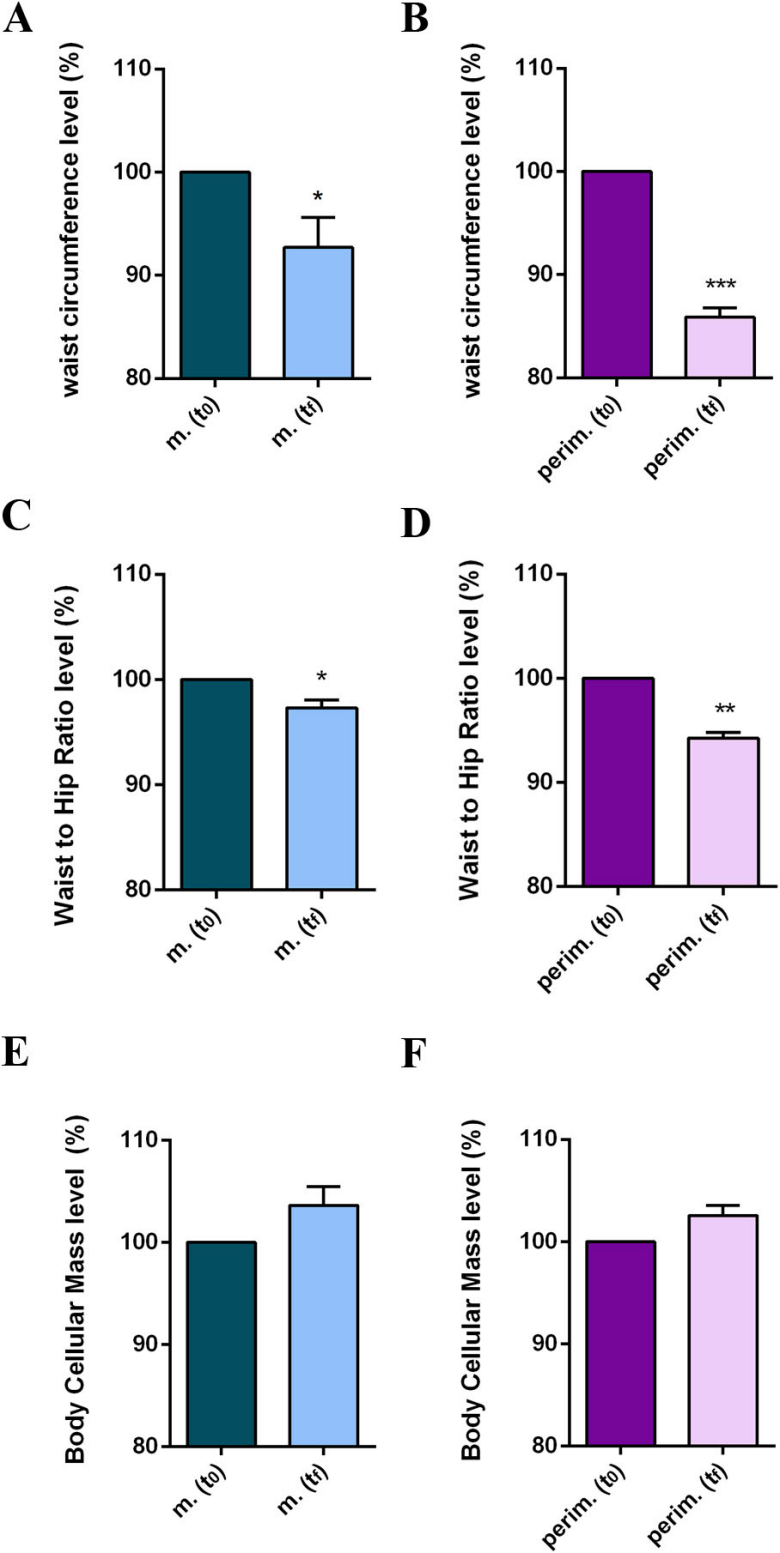
Anthropometric and body composition levels of m. (menopausal) and perim. (perimenopausal) groups at t_0_ (initial evaluation) and t_*f*_ (final follow-up). Waist circumference of m. **(A)** and perim. **(B)** Groups. Waist to hip ratio in m. **(C)** and perim. **(D)** groups. Body cellular mass in m. **(E)** and perim. **(F)** groups. Raw data were analyzed using the paired *t*-test (considering a *p*-value ≤ 0.05 as statistically significant). **(A,B)** 95% CI = [–12,93 to –0,40] and [–16,19 to –8,61], respectively. **(C,D)** 95% CI = [–0,038 to –0,007] and [–0,063 to –0,029], respectively. **(E,F)** 95% CI = [–0,145 to 1,900] and [–0,088 to 1,408], respectively. *p < 0.05, **p < 0.01, ***p < 0.001 vs t_0_.

Since waist circumference (WC) is a key parameter for diagnosing metabolic syndrome, and it is particularly problematic for women in the menopausal phase, we analyzed WC measurements and waist-to-hip ratio (WHR) values (Supplementary Table 5). Additionally, the BCM, as a metabolically active mass index, was also evaluated. Most women achieved a WC below 88 cm, the threshold for diagnosing metabolic syndrome, and a slight decrease in WHR. Additionally, a positive increase in BCM values was observed in nearly all women, higher than 10% in some cases, and slightly positive in others. The statistical analysis of WC (Figures 4A, B) and WHR (Figures 4C, D) parameters confirmed a positive progress, although menopausal women had a less robust reduction than perimenopausal women. No statistically significant differences were obtained in pooled data for BCM at t_*f*_ (Figures 4E, F).

## 4 Discussion

### 4.1 Mediterranean diet and supplementation improve lipid profile in perimenopausal and menopausal women

The findings of this preliminary study, conducted on a convenience sample of peri- and menopausal women, highlight how a personalized dietary intervention based on the MD, supplemented with phytosterols and omega-3 fatty acids, suggests potential for improving the lipid profile, including total TC, LDL-C, HDL-C, and TG levels. Our findings support previous positive claims about the MD, which modulates metabolic and molecular mechanisms that promote health, reducing lipid levels, protecting against oxidative stress, inflammation, and the risk of developing multiple chronic diseases (33, 34). The MD includes fruits, whole grains, legumes, minimally processed plant foods, and low saturated fatty acids. Dried fruits are an excellent source of omega-6 and omega-3 fatty acids, which can improve the lipid profile and reduce the risk of atherosclerosis. In menopausal women, supplementation with omega-3 fatty acids significantly reduced TG levels and slightly elevated HDL-C and LDL-C levels without affecting TC values (35). Our results demonstrate that supplementation with phytosterol and omega-3 fatty acids may contribute to the modulation of the lipid profile, including the TC/HDL-C ratio, in menopausal and perimenopausal women. On the contrary, we also observed a decrease in LDL values, probably due to the diet regimen and the supplementation of phytosterols, thus supporting the efficacy of this nutritional approach. Surprisingly, we observed a considerable increase in HDL-cholesterol. Possibly, among the complexities of the HDL-cholesterol balance, a personalized approach, adherence to the Mediterranean diet style, a correct balance of nutrients, and the use of supplements could play a role. Since adherence to a dietary plan is a critical factor in its success (23), the Mediterranean diet is a suitable choice, as it has been shown to promote long-term adherence and a balanced intake of nutrients (36).

### 4.2 Mediterranean diet and supplementation improve anthropometric and body composition in perimenopausal and menopausal women

Our results showed a reduction in body FM but a modest decrease in BW and BMI. Since BMI does not differentiate between lean and FM and may not be as accurate across different age groups, WC may be a more effective tool for identifying individuals at higher risk of cardiovascular diseases or adverse health outcomes (37). Additionally, it has been reported that reducing visceral fat can be achieved through lifestyle modifications, even without significant BW loss (38). Therefore, it is rational that WC reduction might be a more desirable therapeutic goal than simple weight loss. Our results support this claim, demonstrating a significant WC and WHR decrease. In this context, the MD dietary pattern combined with physical activity has shown promising results in managing obesity and menopausal symptoms (39), promoting weight loss maintenance (40, 41), and preventing weight gain, even without energy restriction (34). In menopausal women, maintaining a balanced diet and engaging in moderate physical activity are crucial for overall health. Recent observations suggest that strict adherence to the MD enables menopausal women to improve body composition and reduce FM while preserving muscle mass (42). Our data confirms a positive trend in metabolically active mass, thereby supporting the benefits of the MD and lifestyle.

Collectively, the data from this study highlight the importance of proper nutritional management during the period leading up to menopause and during the early stages of menopause. The MD dietary and supplement approach suggests potential for improving body composition, anthropometric parameters, and lipid profile when combined with specific hypolipidemic nutraceuticals derived from Mediterranean plant extracts, even in the short term. Symptoms during the perimenopausal and menopausal phases could become disabling disorders and possibly deteriorate health; therefore, the period of menopausal transition could be an optimal window to promote long-term healthiness. Omega-3 fatty acid intakes produce a promising positive impact on several menopause symptoms (43). Moreover, emerging evidence suggests that combining natural bioactive compounds targeting complementary pathways, particularly those containing phytosterols, may offer an effective alternative for managing obesity and improving cardiometabolic health in menopausal women (44). Thus, supporting the general benefit of the supplement approach used in this study.

Considering the significance of all these aspects for women during the menopausal period, these preliminary observations deserve further investigation and a deeper analysis. Although the sample size represents a weakness point of this pilot study, *a priori* power analysis and statistical analysis of the data obtained support the feasibility of our approach and the strength of the results obtained. At the same time, the primary aim of this pilot observational study was to lay the ground for more comprehensive future investigations. Therefore, to improve the validity and reliability of the results, future studies should consider a larger sample size and account for ethnic and cultural variability. Furthermore, future investigations should acknowledge the methodological limitations inherent in the non-standardized interviews conducted in this observational study. Although comparable questions were administered to all participants, this represents a methodological limitation that has to be considered.

During the observational period, adherence to the diet and supplement intake was self-reported and not supervised. Although it is typical in observational studies, this limitation needs to be considered in future deeper analyses. To note that participants were informed about the chance to use their outcomes for research purposes after the conclusion of the study, thereby minimizing the risk of reporting bias. Nevertheless, despite the limitations of this observational study, the approach described here could play a key role in reducing the risk of cardiovascular and metabolic diseases. It encourages natural supplements to manage dyslipidemia when conventional medical therapies are not yet necessary in perimenopausal and menopausal women. Thus, it offers valuable insights for managing mild hypercholesterolemia/hyperlipidemia in the proximity of the first period of menopause.

## Data Availability

The original contributions presented in this study are included in this article/Supplementary material, further inquiries can be directed to the corresponding author.
