# Supplementary material for: Synergistic effects of Mediterranean diet combined with phytosterol-based supplements and omega-3 fatty acids on lipid profiles: a pilot study in menopausal women
Source: Front Nutr. 2025 Sep 11;12:1645102. doi: 10.3389/fnut.2025.1645102 (PMC12460143; doi:10.3389/fnut.2025.1645102)
Supplement: Supplementary file 1 [file Table_1.docx]

Supplementary Material

# Supplementary Tables

**Supplementary Table 1.** Baseline characteristics of the subjects. Average age and BMI values of the m. and perim. groups, along with the percentage of women included in the intervals of benchmark outcomes at the first evaluation by the nutritionist. m. (menopausal), perim. (perimenopausal), BMI (Body Mass Index), TC (total cholesterol), LDL-C (low-density lipoprotein-cholesterol), HDL-C (high-density lipoprotein-cholesterol), TG (triglycerides).

| **Outcomes** | **m. (n=9)** | **perim. (n=5)** |
| --- | --- | --- |
| **average age (years old)** | 60,1 ± 2,7 | 52,8±1,6 |
| **average BMI (kg/m^2^)** | 26,8±3,7 | 27,8±3,7 |
| **BMI** |  |  |
| **healthy range (18,5-24,9 kg/m^2^)** | 33% | 20% |
| **overweight (25-29,9 kg/m^2^)** | 34% | 40% |
| **moderate obesity (30-34,9 kg/m^2^)** | 22% | 40% |
| **severe obesity (35-39.9 kg/m^2^)** | 11% | - |
| **TC** |  |  |
| **moderately high (200-239 mg/dl)** | 11% | 20% |
| **high (≥240 mg/dl)** | 89% | 80% |
| **LDL-C** |  |  |
| **moderately high (130-159 mg/dl)** | 11% | - |
| **high (160-189 mg/dl)** | 45% | 80% |
| **very high (≥190 mg/dl)** | 44% | 20% |
| **HDL-C** |  |  |
| **low (<40 mg/dl)** | 100 % | 100% |
| **TG** |  |  |
| **healthy range (<150 mg/dl)** | 78% | 100% |
| **high (>200 mg/dl)** | 22% | - |

**Supplementary Table 2.** Cholesterol profile. Variation (%) of TC, LDL-C, HDL-C values in m. and perim. women from t_0_ to t_f_ point. m. (menopausal), perim. (perimenopausal), TC (total cholesterol), LDL-C (low-density lipoprotein-cholesterol), HDL-C (high-density lipoprotein-cholesterol), t_0_ (initial evaluation), t_f_ (final follow-up).

| **Group** | **TC (mg/dl)** | | | **LDL-C (mg/dl)** | | | **HDL-C (mg/dl)** | | |
| --- | --- | --- | --- | --- | --- | --- | --- | --- | --- |
| **m./perim.** | **t_0_** | **t_f_** | **t_f_ - t_0_ (%)** | **t_0_** | **t_f_** | **t_f_ - t_0_ (%)** | **t_0_** | **t_f_** | **t_f_ - t_0_ (%)** |
| **m. 1** | 232 | 198 | -15% | 168,2 | 111,2 | -34% | 46 | 76 | +65% |
| **m. 2** | 245 | 213 | -13% | 169,8 | 139,6 | -18% | 56 | 58 | +4% |
| **m. 3** | 264 | 210 | -11% | 193,6 | 134 | -31% | 55 | 63 | +15% |
| **m. 4** | 270 | 234 | -11% | 203,4 | 162,2 | -20% | 54 | 60 | +11% |
| **m. 5** | 284 | 228 | -3% | 211,2 | 177,4 | -16% | 50 | 61 | +22% |
| **m. 6** | 262 | 223 | -15% | 174,6 | 145,4 | -17% | 45 | 65 | +44% |
| **m. 7** | 261 | 207 | -24% | 196,4 | 126 | -36% | 45 | 70 | +56% |
| **m. 8** | 245 | 217 | -26% | 182,4 | 149,4 | -18% | 43 | 54 | +26% |
| **m. 9** | 262 | 222 | -21% | 152 | 128,6 | -15% | 59 | 75 | +27% |
| **perim. 1** | 224 | 170 | -19% | 176,4 | 103 | -42% | 36 | 57 | +58% |
| **perim. 2** | 242 | 180 | -19% | 177,4 | 123 | -31% | 41 | 48 | +17% |
| **perim. 3** | 240 | 190 | -13% | 178,8 | 124,4 | -30% | 45 | 56 | +24% |
| **perim. 4** | 260 | 210 | -11% | 194,8 | 128,4 | -34% | 56 | 72 | +29% |
| **perim. 5** | 243 | 198 | -11% | 182,8 | 120,6 | -34% | 52 | 69 | +33% |

**Supplementary Table 3.** Lipid Profile. Variation (%) of TC/HDL-C and TG values in m. and perim. women from t_0_ to t_f_ point. m. (menopausal), perim. (perimenopausal), TC (total cholesterol), HDL-C (high-density lipoprotein-cholesterol), TG (triglycerides), t_0_ (initial evaluation), t_f_ (final follow-up).

| **Group** | **TC/HDL-C** | | | **TG (mg/dl)** | | |  |
| --- | --- | --- | --- | --- | --- | --- | --- |
| **m./perim.** | **t_0_** | **t_f_** | **t_f_ - t_0_ (%)** | **t_0_** | **t_f_** | **t_f_ - t_0_ (%)** | |
| **m. 1** | 5,04 | 2,61 | -48% | 89 | 54 | -39% | |
| **m. 2** | 4,38 | 3,67 | -16% | 96 | 77 | -20% | |
| **m. 3** | 4,8 | 3,33 | -31% | 77 | 65 | -16% | |
| **m. 4** | 5 | 3,9 | -22% | 63 | 59 | -6% | |
| **m. 5** | 5,68 | 3,74 | -34% | 114 | 68 | -40% | |
| **m. 6** | 5,82 | 3,43 | -41% | 212 | 63 | -70% | |
| **m. 7** | 5,8 | 2,96 | -49% | 98 | 55 | -44% | |
| **m. 8** | 5,7 | 4,02 | -29% | 98 | 68 | -31% | |
| **m. 9** | 4,44 | 2,96 | -33% | 255 | 92 | -64% | |
| **perim. 1** | 6,22 | 2,98 | -52% | 58 | 50 | -14% | |
| **perim. 2** | 5,9 | 3,75 | -36% | 118 | 45 | -62% | |
| **perim. 3** | 5,33 | 3,39 | -36% | 53 | 48 | -9% | |
| **perim. 4** | 4,64 | 2,92 | -37% | 46 | 48 | +4% | |
| **perim. 5** | 4,67 | 2,87 | -39% | 41 | 42 | +2% | |

**Supplementary Table 4.** Anthropometric measurements and body composition. Variation (%) of BW (kg), BMI (kg/m^2^), and FM (kg) values in m. and perim. women from t_0_ to t_f_ point. m. (menopausal), perim. (perimenopausal), BW (body weight), BMI (Body Mass Index), FM (fat mass), t_0_ (initial evaluation), t_f_ (final follow-up).

| **Group** | **BW (kg)** | | | **BMI (kg/m^2^)** | | | **FM (kg)** | | |
| --- | --- | --- | --- | --- | --- | --- | --- | --- | --- |
| **m./perim.** | **t_0_** | **t_f_** | **t_f_ - t_0_ (%)** | **t_0_** | **t_f_** | **t_f_ - t_0_ (%)** | **t_0_** | **t_f_** | **t_f_ - t_0_ (%)** |
| **m. 1** | 62,5 | 59,8 | -4% | 22,5 | 21,2 | -6% | 16,3 | 11,8 | -28% |
| **m. 2** | 67,2 | 65,5 | -3% | 24,1 | 23,5 | -2% | 21,5 | 19,4 | -10% |
| **m. 3** | 79,1 | 80,6 | +2% | 29,1 | 29,6 | +2% | 28 | 26,5 | -5% |
| **m. 4** | 76,9 | 72,4 | -6% | 26,3 | 24,8 | -6% | 28 | 22,7 | -19% |
| **m. 5** | 56,3 | 55,5 | -1% | 24,4 | 24 | -2% | 18,4 | 17,6 | -4% |
| **m. 6** | 73,9 | 68,5 | -7% | 30,4 | 28,3 | -7% | 29,7 | 24,1 | -19% |
| **m. 7** | 70,5 | 68,2 | -3% | 30,5 | 29,5 | -3% | 24,2 | 22 | -9% |
| **m. 8** | 97 | 85,5 | -12% | 37,4 | 33 | -12% | 40,1 | 30 | -25% |
| **m. 9** | 70,1 | 63,9 | -9% | 29,6 | 27 | -9% | 25,6 | 19,2 | -25% |
| **perim. 1** | 74,6 | 65,2 | -13% | 30,7 | 26,8 | -13% | 30,5 | 20,7 | -32% |
| **perim. 2** | 67,8 | 61,8 | -9% | 27,2 | 24,8 | -9% | 30,9 | 23,7 | -23% |
| **perim. 3** | 70,3 | 62,8 | -11% | 26,1 | 23,4 | -10% | 23 | 16,2 | -30% |
| **perim. 4** | 94 | 83,1 | -12% | 32,2 | 28,4 | -12% | 38,6 | 29,7 | -23% |
| **perim. 5** | 64,7 | 61,8 | -4% | 22,9 | 21,9 | -4% | 19,1 | 16,1 | -16% |

**Supplementary Table 5.** Anthropometric values and body composition. Variation (%) of values for the WC (cm), WHR, and BCM (kg) of m. and perim. women from t_0_ to t_f_ point. m. (menopausal), perim. (perimenopausal), WC (waist circumference), WHR (waist-to-hip ratio), and BCM (body composition mass), t_0_ (initial evaluation), t_f_ (final follow-up).

| **Group** | **WC (cm)** | | | **WHR** | | | **BCM (kg)** | | |
| --- | --- | --- | --- | --- | --- | --- | --- | --- | --- |
| **m./perim.** | **t_0_** | **t_f_** | **t_f_ - t_0_ (%)** | **t_0_** | **t_f_** | **t_f_ - t_0_ (%)** | **t_0_** | **t_f_** | **t_f_ - t_0_ (%)** |
| **m. 1** | 73 | 72 | -1% | 0,77 | 0,78 | +1% | 23,6 | 26 | +10% |
| **m. 2** | 83 | 90 | +8% | 0,83 | 0,81 | -2% | 23,9 | 27,5 | +15% |
| **m. 3** | 91 | 89 | -2% | 0,84 | 0,82 | -2% | 26,7 | 27,7 | +4% |
| **m. 4** | 83 | 75 | -10% | 0,81 | 0,77 | -5% | 25 | 24,3 | -3% |
| **m. 5** | 74 | 66 | -11% | 0,76 | 0,73 | -4% | 20,4 | 20,6 | +1% |
| **m. 6** | 96 | 83 | -14% | 0,91 | 0,86 | -5% | 23,8 | 24,2 | +2% |
| **m. 7** | 89 | 85 | -4% | 0,88 | 0,87 | -1% | 25,3 | 25,3 | 0% |
| **m. 8** | 96 | 87 | -9% | 0,75 | 0,73 | -3% | 32,7 | 32,9 | +1% |
| **m. 9** | 97 | 75 | -23% | - | - | - | 24,1 | 24,9 | +3% |
| **perim. 1** | 93 | 77 | -17% | 0,85 | 0,79 | -7% | 25,9 | 26,1 | +1% |
| **perim. 2** | 96 | 82 | -15% | 0,91 | 0,85 | -7% | 24,3 | 25,2 | +4% |
| **perim. 3** | 78 | 67 | -14% | 0,72 | 0,68 | -6% | 27,4 | 28,8 | +5% |
| **perim. 4** | 99 | 86 | -13% | 0,78 | 0,75 | -4% | 28,6 | 28,5 | 0% |
| **perim. 5** | 69 | 61 | -12% | 0,7 | 0,66 | -6% | 25,3 | 26,2 | +4% |
